# Supplementary material for: Chemical fixation creates nanoscale clusters on the cell surface by aggregating membrane proteins
Source: Commun Biol. 2022 May 20;5:487. doi: 10.1038/s42003-022-03437-2 (PMC9122943; doi:10.1038/s42003-022-03437-2)
Supplement: Supplementary file 2 — Supplementary Information [file 42003_2022_3437_MOESM2_ESM.pdf]

Supplementary Information for

Chemical fixation creates nanoscale clusters on the cell surface by aggregating membrane proteins.

Takehiko Ichikawa[1]*, Dong Wang[1,2], Keisuke Miyazawa[1,3], Kazuki Miyata[1,3], Masanobu Oshima[1,2]*, Takeshi Fukuma[1,3]*

[1]Nano Life Science Institute (WPI-NanoLSI), Kanazawa University, Kanazawa, 920-1192, Japan, [2]Division of Genetics, Cancer Research Institute, Kanazawa University, Kanazawa, 920-1192, Japan, [3]Faculty of Frontier Engineering, Kanazawa University, Kanazawa, 920-1192, Japan

** Author for correspondence*
Takehiko Ichikawa, Ph.D.
Nano Life Science Institute, Kanazawa University,
Kanazawa, 920-1192, Japan
E-mail: tichikawa@staff.kanazawa-u.ac.jp

Masanobu Oshima, PhD.
Nano Life Science Institute, Kanazawa University,
Kanazawa, 920-1192, Japan
Division of Genetics, Cancer Research Institute,
Kanazawa University, Kanazawa, 920-1192, Japan
E-mail: oshimam@staff.kanazawa-u.ac.jp

Takeshi Fukuma, PhD
Nano Life Science Institute, Kanazawa University,
Kanazawa, 920-1192, Japan
Faculty of Frontier Engineering, Kanazawa University,
Kanazawa, 920-1192, Japan
E-mail: fukuma@staff.kanazawa-u.ac.jp

Supplementary figures

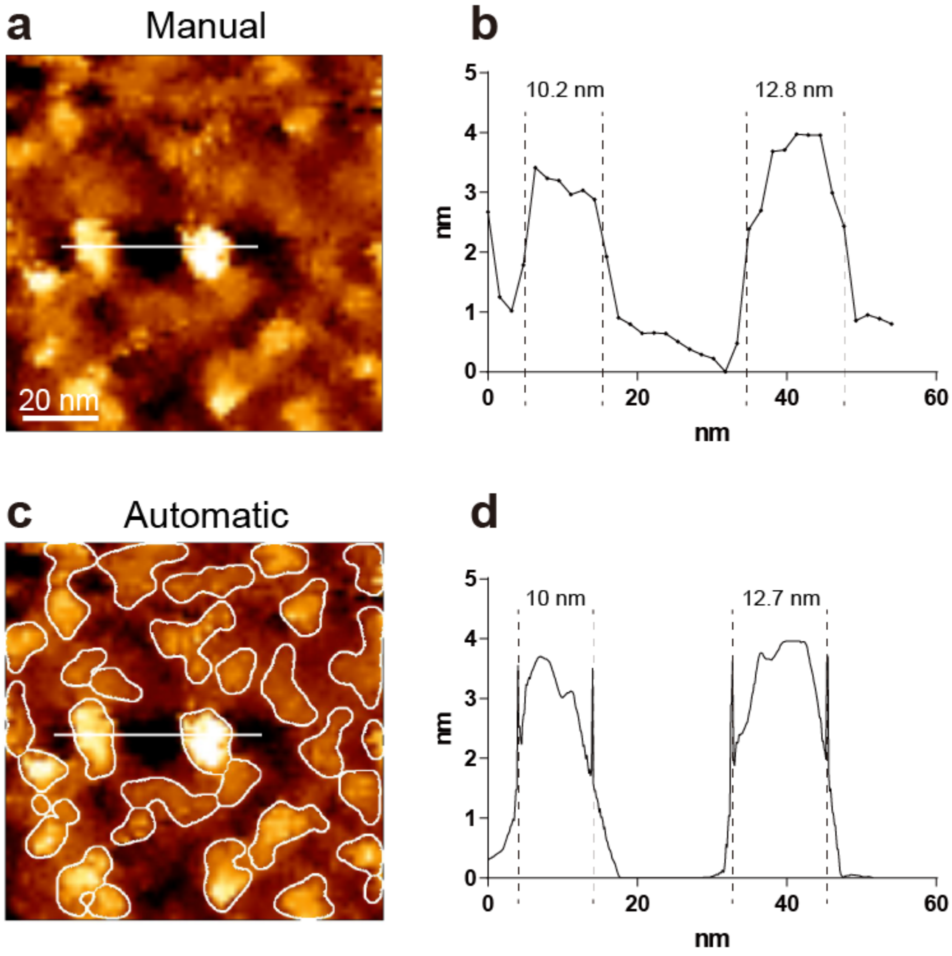

Supplementary Figure 1. Automatic tool for recognizing protrusions on the cell surface shown by the AFM image.

(a) Original AFM image at a 100 × 100 nm scale. Scale bar: 20 nm. (b) Height profile along the line shown in a. The full width at half maximum (FWHM) was manually measured as 10.2 nm (left) and 12.8 nm (right). (c) The boundary of the automatically recognized area at the half-height of each protrusion on the AFM image was superimposed using the custom-made script. White contour lines show the boundary of the recognized area. (d) Height profile along the line shown in c. The full width at half maximum (FWHM) was measured as 10 nm (left) and 12.7 nm (right).

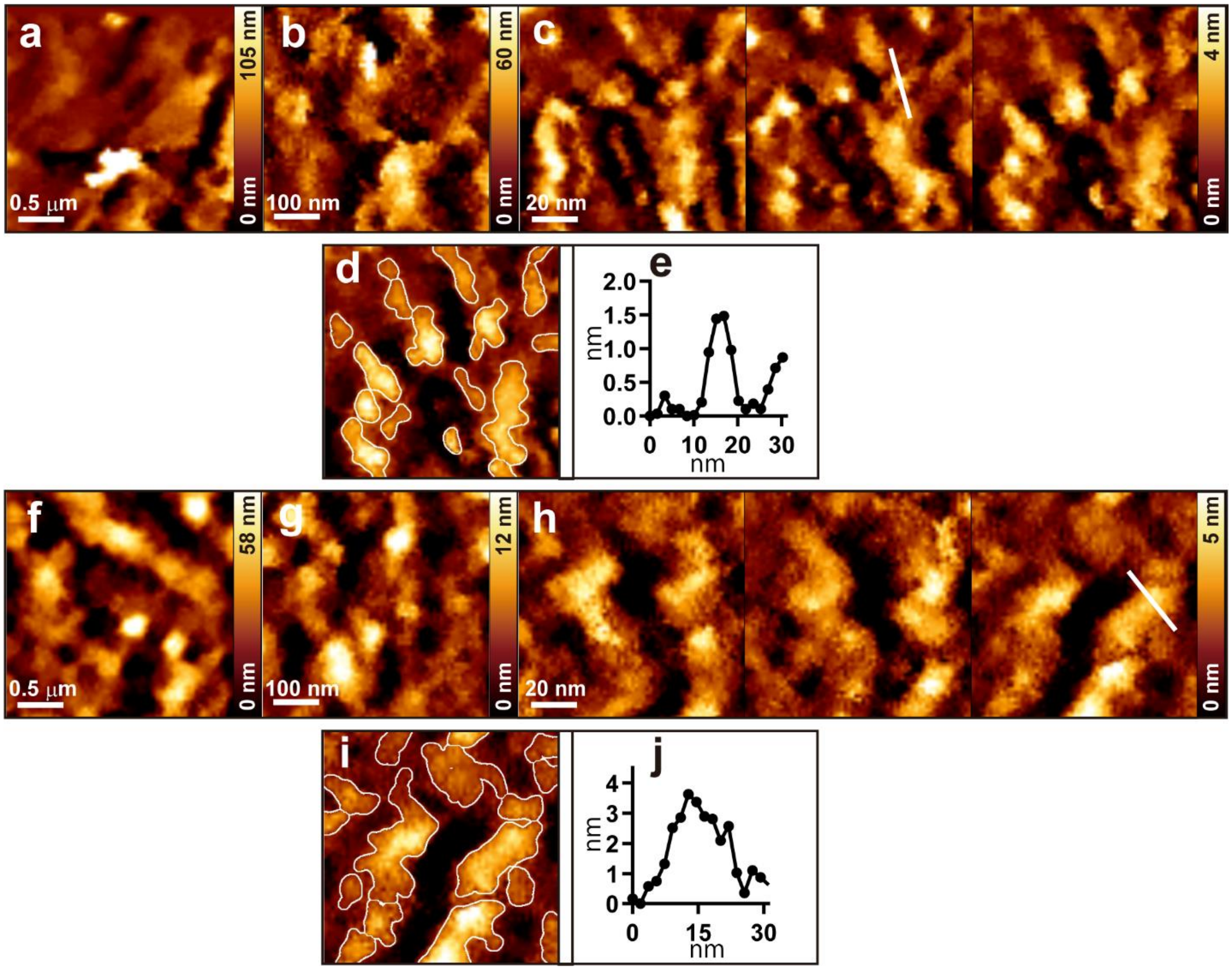

Supplementary Figure 2. An additional result of AFM measurement of DLD-1 cell surface using AC40 cantilever.

(a-e) Live imaging of DLD-1 cell surface using AC40TS-C2 cantilever (Olympus, spring constant approximately 0.1 N/m). (a) 2.5 × 2.5 µm scale image. (b) 0.5 × 0.5 µm scale image. (c) Three consecutive images of 100 × 100 nm scale acquired every 2 min. (d) Superimposed image of the third image in c with the boundary of the recognized protrusion area (white line). (e) Height profile along the line in c. FWHM is 6.2 nm. (f-j) AFM image of DLD-1 cell surface after treatment of 2% GA. (f) 2.5 × 2.5 µm scale image. (g) 0.5 × 0.5 µm scale image. (h) Three consecutive images of 100 × 100 nm scale. (i) Superimposed image of the third image in h with the boundary of the recognized protrusion area (white line). (j) Height profile along the line in h. FWHM is 15.3 nm.

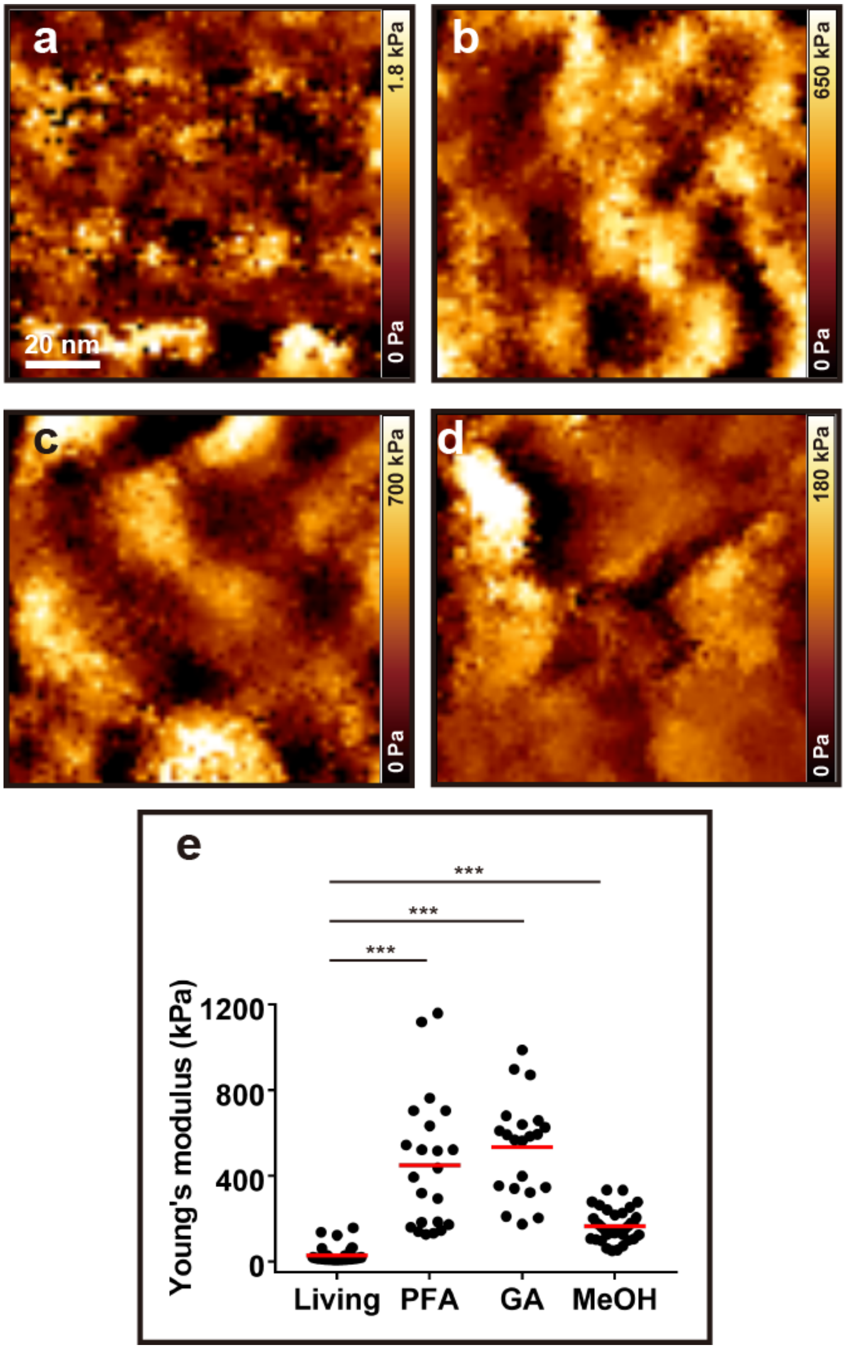

Supplementary Figure 3. Young's modulus of the living or fixed cell surface.
(a) Young's modulus map of the living cell surface at a 100 × 100 nm scale. Scale bar: 20 nm. (b) After treatment with 4% PFA. (c) After treatment with 2% GA. (d) After treatment with cold 100% MeOH. (e) Young's modulus distributions on the cell surfaces of living, PFA, GA, and MeOH treated cells. Red bars indicate mean values. Mean values (± SEM) are living: 27.21 ± 5.43 (n = 41), PFA: 449 ± 65.46 (n = 22), GA: 534.8 ± 49.7 (n = 21) and MeOH: 165.3 ± 11.64 (n = 38) kPa. Asterisks (***) indicate statistical significance ($p < 0.001$, two-sided t-test).

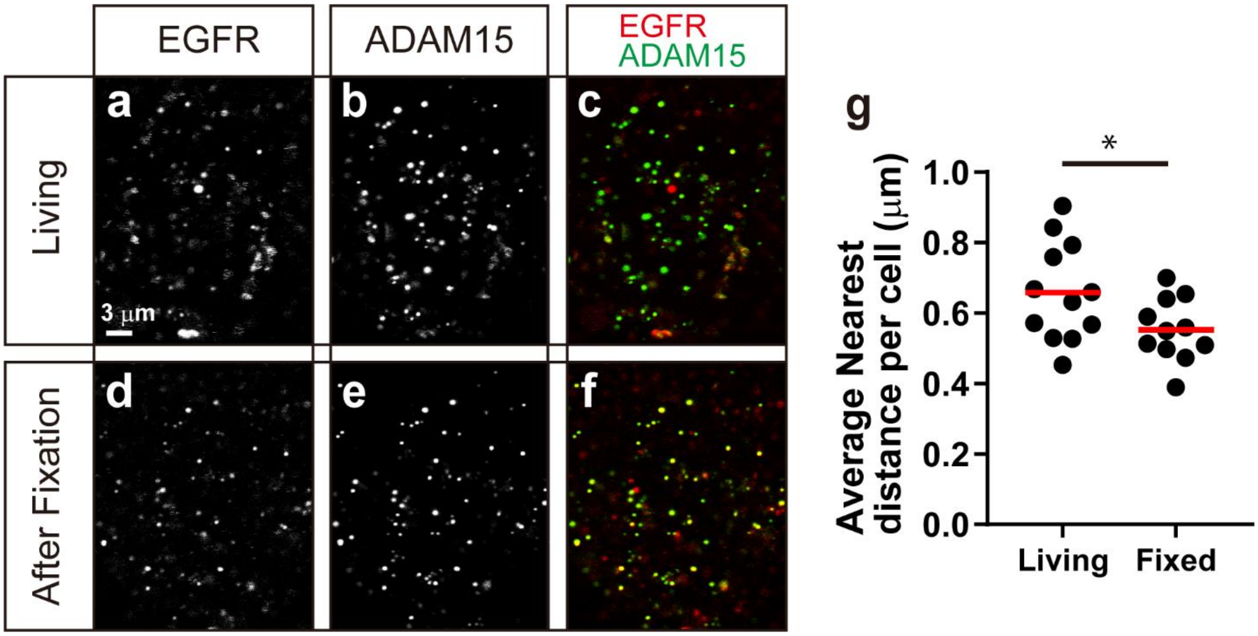

Supplementary Figure 4. Nearest distance change of EGFR and ADAM15 after fixation.
(a) EGFR image of a living cell. (b) ADAM15 image of the same cell as a. (c) Superimposed image of EGFR (a, red) and ADAM15 (b, green). (d) EGFR image after 2% GA treatment. (e) ADAM15 image after 2% GA treatment of the same cell as d. (f) Superimposed image of EGFR (d, red) and ADAM15 (e, green). (g) Distribution of the average nearest distance per cell ($n = 12$ for living cell, $n = 11$ for fixed cell). Red lines indicate the mean of the distribution. Asterisks (*) denote statistical significance ($p < 0.05$, two-sided t-test).

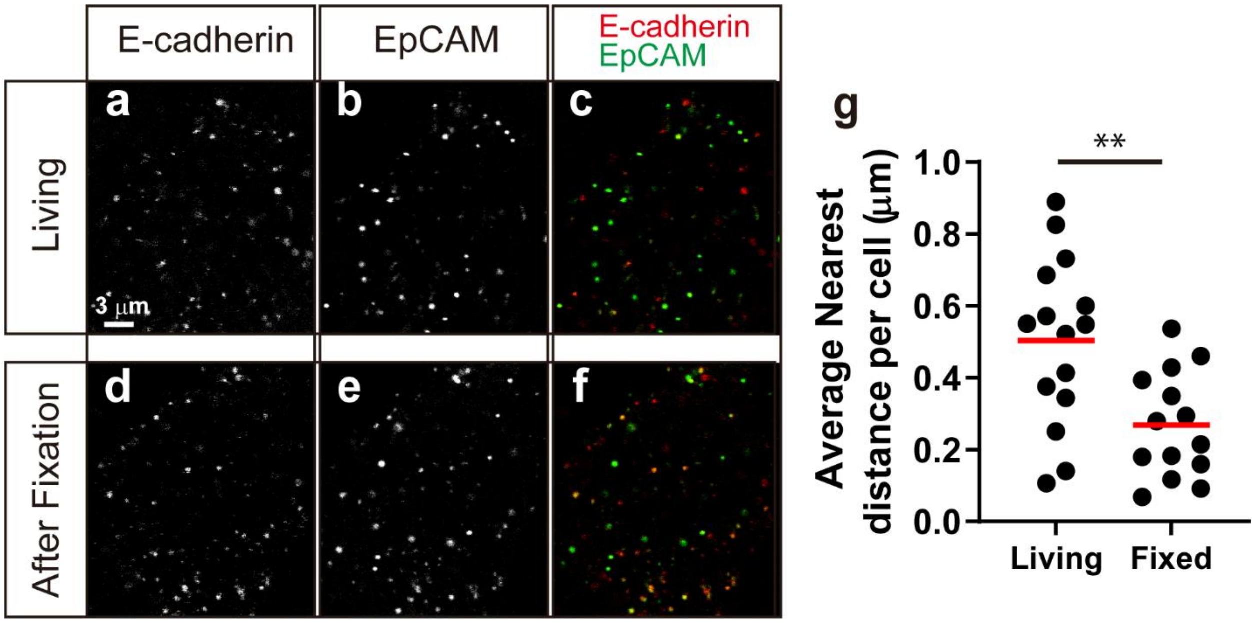

Supplementary Figure 5. Nearest distance of two kinds of membrane proteins using HeLa cell.

(a) E-cadherin image of a living cell. (b) EpCAM image of the same cell as a. (c) Superimposed image of E-cadherin (a, red) and EpCAM (b, green). (d) E-cadherin image after 4% PFA treatment. (e) EpCAM image after 4% PFA treatment of the same cell as d. (f) Superimposed image of E-cadherin (d, red) and EpCAM (e, green). (g) Distribution of the average nearest distance per cell (n = 15 for living cell, n = 14 for fixed cell). Red lines indicate the mean values. Asterisks (**) denote statistical significance ($p < 0.01$, two-sided t-test).

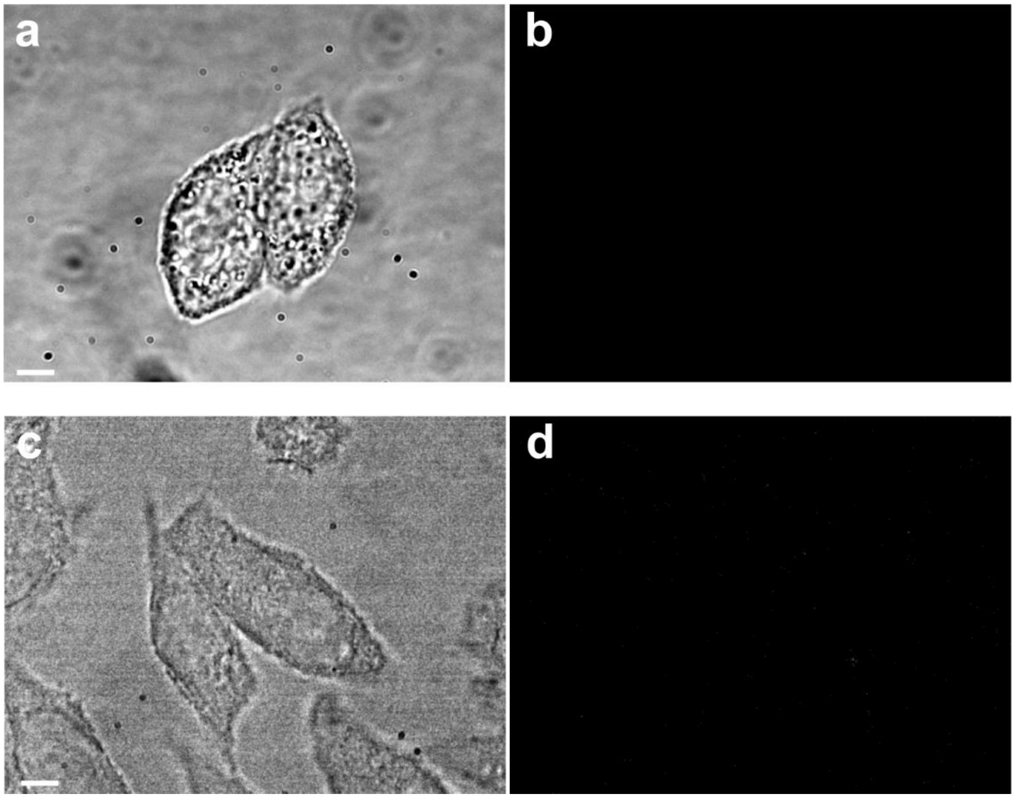

Supplementary Figure 6. Controll experiment of no laballing cell.

(a) Bright-field image of the living DLD-1 cell. Scale bar is 5 μm. (b) Fluorescence image of the same cell as a. (c) Bright-field image of the DLD-1 cell fixed by 4% PFA. Scale bar is 5 μm. (d) Fluorescence image of the same cell as c.

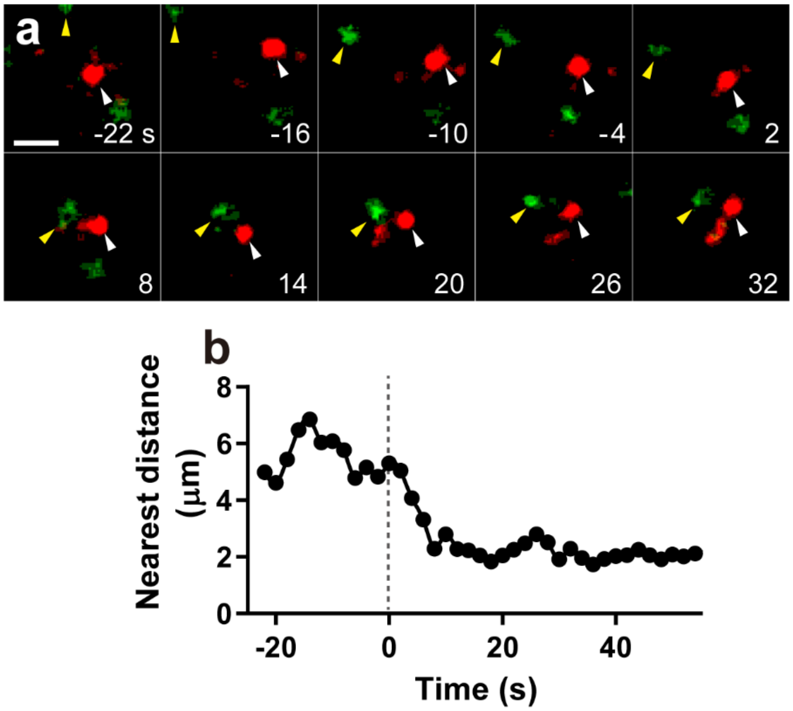

Supplementary Figure 7. Time series during fixation using EGFR and ADAM15.

(a) Time series during fixation. EGFR (red) and ADAM15 (green) are stained. Arrowheads of the same colour indicate the same molecules. The scale bar is 3 μm. 2% GA was added at 0 sec. (b) Time-lapse change of the distance between indicated EGFR and ADAM15 molecules. The dotted line indicates the time point of GA addition.

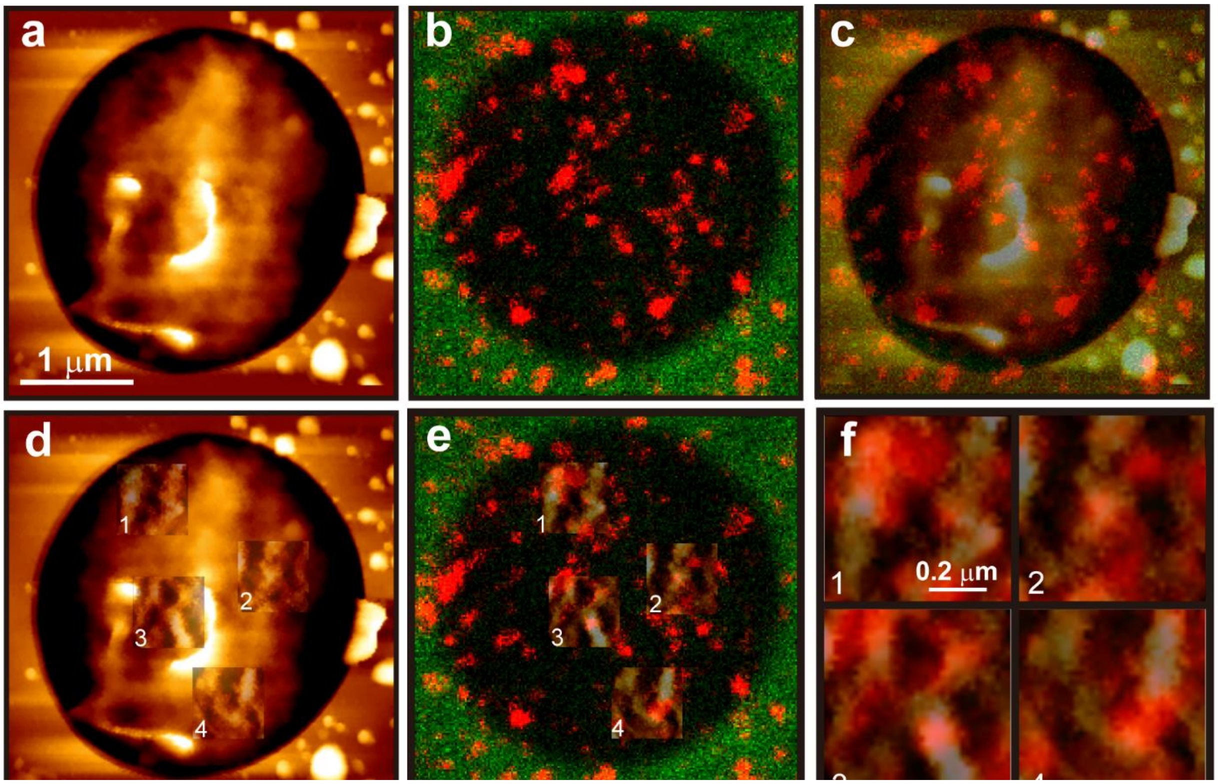

Supplementary Figure 8. Correspondence of AFM and STED image of EGFR.
(a) AFM image of PFA fixed DLD-1 cell cultured on 3 μm MPM. (b) STED image of the same position and scale depicted on a. Red spots indicate the localization of EGFR. The green colour shows the MPM surface. (c) Superimposed image of AFM and fluorescence images. (d) Superimposed image of cropped and contrast adjusted AFM image and original AFM image. (e) Superimposed image of cropped AFM image and STED image. (f) Magnified overlayed image of e. Numbers correspond in e.
