# Supplementary material for: Chemical fixation creates nanoscale clusters on the cell surface by aggregating membrane proteins
Source: Commun Biol. 2022 May 20;5:487. doi: 10.1038/s42003-022-03437-2 (PMC9122943; doi:10.1038/s42003-022-03437-2)
Supplement: Supplementary file 3 — Reporting Summary [file 42003_2022_3437_MOESM3_ESM.pdf]

nature portfolio

Corresponding author(s): Takehiko Ichikawa

Last updated by author(s): Apr 18, 2022

Reporting Summary

Nature Portfolio wishes to improve the reproducibility of the work that we publish. This form provides structure for consistency and transparency in reporting. For further information on Nature Portfolio policies, see our Editorial Policies and the Editorial Policy Checklist.

Statistics

For all statistical analyses, confirm that the following items are present in the figure legend, table legend, main text, or Methods section.

n/a	Confirmed	
☐	☒	The exact sample size (n) for each experimental group/condition, given as a discrete number and unit of measurement
☐	☒	A statement on whether measurements were taken from distinct samples or whether the same sample was measured repeatedly
☐	☒	The statistical test(s) used AND whether they are one- or two-sided<br>*Only common tests should be described solely by name; describe more complex techniques in the Methods section.*
☒	☐	A description of all covariates tested
☐	☒	A description of any assumptions or corrections, such as tests of normality and adjustment for multiple comparisons
☐	☒	A full description of the statistical parameters including central tendency (e.g. means) or other basic estimates (e.g. regression coefficient) AND variation (e.g. standard deviation) or associated estimates of uncertainty (e.g. confidence intervals)
☐	☒	For null hypothesis testing, the test statistic (e.g. F, t, r) with confidence intervals, effect sizes, degrees of freedom and P value noted<br>*Give P values as exact values whenever suitable.*
☒	☐	For Bayesian analysis, information on the choice of priors and Markov chain Monte Carlo settings
☒	☐	For hierarchical and complex designs, identification of the appropriate level for tests and full reporting of outcomes
☒	☐	Estimates of effect sizes (e.g. Cohen's d, Pearson's r), indicating how they were calculated

Our web collection on statistics for biologists contains articles on many of the points above.

Software and code

Policy information about availability of computer code

Data collection	NanoWizard control software (JPK, Bruker) for AFM imaging, Imspector (Abberior Instruments) for confocal and STED imaging
Data analysis	JPK data processing software (Bruker) and custom software programmed in Matlab (Simulink) for image analysis, and Prism 7 (GraphPad Software) for data visualization.

For manuscripts utilizing custom algorithms or software that are central to the research but not yet described in published literature, software must be made available to editors and reviewers. We strongly encourage code deposition in a community repository (e.g. GitHub). See the Nature Portfolio guidelines for submitting code & software for further information.

Data

Policy information about availability of data

All manuscripts must include a data availability statement. This statement should provide the following information, where applicable:

- Accession codes, unique identifiers, or web links for publicly available datasets
- A description of any restrictions on data availability
- For clinical datasets or third party data, please ensure that the statement adheres to our policy

The source data used in this study is supplied in FigShare: 10.6084/m9.figshare.19609362. Correspondence and requests for materials should be addressed to Takehiko Ichikawa (tichikawa@staff.kanazawa-u.ac.jp).

Field-specific reporting

Please select the one below that is the best fit for your research. If you are not sure, read the appropriate sections before making your selection.

☒ Life sciences ☐ Behavioural & social sciences ☐ Ecological, evolutionary & environmental sciences

For a reference copy of the document with all sections, see nature.com/documents/nr-reporting-summary-flat.pdf

Life sciences study design

All studies must disclose on these points even when the disclosure is negative.

Sample size	We examined more than 3 times for each experiment. We took more than 100 images from 5 cells for AFM and took more than 10 images from 10 cells for fluorescence imaging. We did 5 times for Figure 3n and 4 times for Figure 4.
Data exclusions	We did not chose unhealthy cell and did not exclude any acquired data.
Replication	We confirmed the reproducibility.
Randomization	We chose cells randomly.
Blinding	Our experiments are not in this case.

Reporting for specific materials, systems and methods

We require information from authors about some types of materials, experimental systems and methods used in many studies. Here, indicate whether each material, system or method listed is relevant to your study. If you are not sure if a list item applies to your research, read the appropriate section before selecting a response.

Materials & experimental systems

n/a	Involved in the study	
☐	☒	Antibodies
☐	☒	Eukaryotic cell lines
☒	☐	Palaeontology and archaeology
☒	☐	Animals and other organisms
☒	☐	Human research participants
☒	☐	Clinical data
☒	☐	Dual use research of concern

Methods

n/a	Involved in the study	
☒	☐	ChIP-seq
☒	☐	Flow cytometry
☒	☐	MRI-based neuroimaging

Antibodies

Antibodies used	Primary antibodies: E-cadherin antibody (ab40772, Abcam, Lot No. GR3209210-17), EpCAM antibody (14-9326-82, Thermo Fisher Scientific, Lot No. 2190445), EGFR antibody (ab52894, Abcam, Lot No. GR3214138-3), ADAM15 antibody (MAB935-SP, R & D Systems, Lot No. DXQ022101A), Secondary antibodies: STAR RED goat anti-rabbit IgG (STRED-1002, Abberior, Lot No. 00209JR-5), STAR ORANGE goat anti-mouse IgG (STAR ORANGE-1001, Abberior, Lot No. 00422JR-7).
Validation	E-cadherin antibody is validated by western blot (for human cells, PMID: 28601643), immunofluorescence (for human cells, PMID: 34434285) and flow cytometry (for human cells, PMID: 32726133). EpCAM antibody is validated by western blot (for human cells, PMID: 34022398), immunofluorescence (for human cells, PMID: 32722876) and flow cytometry (for human cells, PMID: 30951821). EGFR antibody is validated by western blot (for human cells, PMID: 32732965), immunofluorescence (for human cells, PMID: 31345374) and flow cytometry (for human cells, PMID: 29045902). ADAM15 antibody is validated by western blot (for human cells, PMID: 22505472), immunofluorescence (for human cells, PMID: 22544741) and flow cytometry (for human cells, PMID: 25333931).

Eukaryotic cell lines

Policy information about cell lines

Cell line source(s)	DLD-1 was supplied by the Cell Resource Center for Biomedical Research, Tohoku University, Japan.
Authentication	None of the cell lines used were authentificated.
Mycoplasma contamination	The cell lines were not tested for mycoplasma contamination.

Commonly misidentified lines<br>(See ICLAC register)	The cells used are not on the list of Commonly misidentified lines.
